# Gentamicin‐induced sensorineural auditory loss in healthy adult horses

**DOI:** 10.1111/jvim.16221

**Published:** 2021-07-28

**Authors:** Monica R. Aleman, Alexander True, Rebeca Scalco, Chelsea M. Crowe, Lais R. R. Costa, Munashe Chigerwe

**Affiliations:** ^1^ Department of Medicine and Epidemiology School of Veterinary Medicine, University of California Davis California USA; ^2^ William R. Pritchard Veterinary Medical Teaching Hospital School of Veterinary Medicine, University of California Davis California USA

**Keywords:** aminoglycosides, BAEP, deafness, hearing, ototoxicity, vestibular

## Abstract

**Background:**

Irreversible sensorineural auditory loss has been reported in humans treated with aminoglycosides but not in horses.

**Objective:**

Investigate if auditory loss occurs in horses treated using the recommended IV daily dosage of gentamicin for 7 consecutive days.

**Animals:**

Ten healthy adult horses (7‐15 years; females and males, 5 each).

**Methods:**

Prospective study. Physical and neurological examinations and renal function tests were performed. Gentamicin sulfate was administered at a dosage of 6.6 mg/kg via the jugular vein on alternating sides for 7 days. Gentamicin peak and trough concentrations were measured. Horses were sedated using detomidine hydrochloride IV to perform brainstem auditory evoked responses (BAER) before the first dose, immediately after the last dose, and 30 days after the last dose. Peaks latencies, amplitudes, and amplitude ratios were recorded. Data from the second and last BAER were compared to results at baseline. Bone conduction was performed to rule out conduction disorders.

**Results:**

Seven horses had auditory loss: complete bilateral (N = 1), complete unilateral (N = 2), and partial unilateral (N = 4). Based on physical examination and BAER results, sensorineural auditory loss was suspected. Absent bone conduction ruled out a conduction disorder and further supported sensorineural auditory loss in horses with completely absent BAER. Auditory dysfunction was reversible in 4 of 7 horses.

**Conclusions and Clinical Importance:**

Gentamicin at recommended doses may cause sensorineural auditory loss in horses that might be irreversible. Follow‐up studies are needed to investigate if other dosing protocols present a similar risk.

**Abbreviations**
AG: aminoglycoside
BAER: brainstem auditory evoked response
BC: bone conduction
C2: second cervical vertebra
GENT: gentamicin
IPI: interpeak interval
LM: left mastoid
nHL: normal hearing level
RM: right mastoid
V: vertex

## 1 INTRODUCTION

Auditory loss attributed to temporohyoid osteoarthropathy, otitis, brainstem disease, trauma, congenital sensorineural deafness in American Paint horses, and old age has been reported in horses.^1^, ^2^, ^3^, ^4^, ^5^, ^6^ Causes of auditory loss in foals include congenital sensorineural deafness, hypoxic ischemic encephalopathy, bilirubin encephalopathy, prematurity, sepsis, brainstem disease, trauma, and otitis.^2^ Although aminoglycoside‐induced auditory loss has been suspected in horses, it has not been conclusively identified.^1^, ^2^ A study investigating the effects of gentamicin (GENT) administered IM at a dosage of 5 mg/kg q8h in 6 adult ponies for 7 and 14 days (3 ponies in each protocol) identified no alterations in auditory function.^7^ The route and dose of GENT used in that study were different than currently used protocols in equine medicine.^7^, ^8^, ^9^ Aminoglycosides (AG) such as GENT are commonly used in conjunction with beta‐lactams in horses for an average duration of 7 days, depending on the type of infection.^10^ However, in some critically ill‐patients, AG administration might be extended beyond 30 days.^11^ Similar to suspected auditory loss, an association between GENT administered IV and vestibular disease was suspected in a horse treated for pleuropneumonia.^12^ More commonly, vestibular disease in horses can result from trauma, temporohyoid osteoarthropathy, otitis media or interna, meningoencephalomyelitis, brainstem disease, equine protozoal myeloencephalitis, mass occupying lesions, and toxic causes (drugs, lolitrem B), among other causes.^4^, ^13^, ^14^, ^15^, ^16^, ^17^

Several drugs reported to have ototoxic effects in humans that also are used in equine medicine include AG, macrolides, vancomycin, cisplatin, furosemide, and salicylates.^18^ Aminoglycoside ototoxicity resulting in cochleo‐ and vestibulotoxicity has been well‐documented in humans, dogs, and cats.^19^, ^20^, ^21^, ^22^, ^23^, ^24^ In some cases, AG‐induced cochleotoxicity also may manifest as tinnitus.^19^ Tinnitus has been reported rarely in horses the etiology of which was unknown.^25^ Aminoglycosides have been used effectively for the treatment of Gram negative bacterial infections.^26^ Nine AG been approved for medical use: streptomycin, neomycin, tobramycin, kanamycin, paromomycin, spectinomycin, netilmicin, gentamicin, and amikacin.^26^ Gentamicin and amikacin are the AG most commonly used in equine medicine,^27^ and both reportedly are potentially ototoxic.^18^ Such an association has not been fully investigated in horses at currently used routes and dosages of GENT administration. Our objective was to investigate auditory and vestibular function in healthy adult horses treated with GENT IV at the approved dosage for 7 consecutive days. Vestibular and auditory function were evaluated by neurological examination and brainstem auditory‐evoked response (BAER), respectively. We hypothesized that GENT administration would alter auditory function in horses.

## 2 MATERIALS AND METHODS

### 2.1 Animals

Ten healthy adult horses from a research herd were selected based on availability for the study. These horses consisted of 5 mares of Warmblood, Standardbred, and Quarter horse related breeds with ages ranging from 7 to 14 years. Five geldings of Quarter horse, Thoroughbred, and mixed breeds with ages ranging from 9 to 15 years. Horses had normal physical examination findings. Neurological examination to assess vestibular and auditory function was performed by a board‐certified neurologist (Aleman) before administering the first dose, after the last dose (day 7), and 30 days after discontinuation of GENT administration. Vestibular function was evaluated by observation of the presence or absence of nystagmus, head and body posture, gait, and reaction to blindfolding. Auditory function was evaluated by the horses' response to environmental auditory stimuli, and clapping of hands by the examiner. Horses' weights were recorded for the calculation of detomidine hydrochloride (Dormosedan, Zoetis, Inc, Kalamazoo, MI) and GENT sulfate (VetOne, Bose, ID) dosages. Weights ranged from 500 to 666 kg (586 ± 52 kg). All horses were housed in covered independent pens at the research facility, fed grass hay, provided free access to water, and monitored daily by the investigators. The study was approved by the University of California at Davis Institutional Animal Care and Use Committee #22043.

### 2.2 Clinical laboratory testing

Blood samples from the jugular vein were collected and serum separated for analysis of renal function tests as part of health status evaluation before the first and last dose of GENT. The serum biochemical panel included concentrations of creatinine, blood urea nitrogen, total protein, albumin, bicarbonate, electrolytes (sodium, potassium, chloride, phosphorus, calcium), and calculated anion gap.

### 2.3 Gentamicin protocol

A dose of GENT sulfate of 6.6 mg/kg was calculated and administered IV through the jugular vein once a day in the morning for 7 consecutive days. The injection side was alternated daily. Blood samples were collected in a red top tubes (Monoject, Covidien, Mansfield, MA) from the jugular vein before administration of the last dose of GENT and 1 hour postadministration to assess trough and peak concentrations, respectively, using radioimmunoassay.

### 2.4 Sedation protocol

The dose of detomidine hydrochloride IV ranged from 0.01 to 0.02 mg/kg (5‐13 mg total) depending on the individual horse's behavior and demeanor. Horses were placed in standing stocks before placement of earphones and SC electrodes. The extent of sedation was considered adequate for the study when horses appeared quiet with their heads low and supported by the stocks.

### 2.5 Brainstem auditory evoked potentials

All BAER studies were performed at our institution's research facility. Brainstem auditory evoked response testing was done according to our UCD protocol using a Nicolet VikingQuest (Nicolet Viasys Healthcare, Madison, WI). Three BAER studies were performed: before the first dose, after the last dose (day 7), and 30 days after discontinuation of GENT administration (day 37). Before placement of insert earphones, the ears were gently cleaned using a dampened gauze. Disposable insert earphones (Etymotic Research, Inc, ER3‐14A, Elk Grove Village, IL) were placed in both ears, and SC 24″ wire needle electrodes (Natus, F‐E2‐24 Grass Platinum Subdermal Needle Electrodes, Middletown, WI) were placed in the scalp. Insert earphones were digitally placed in the external ear canal. The earphones were inspected at the end of each study to rule out the presence of debris or cerumen that could obstruct the conduction of sound. Subcutaneous needle electrodes (n = 4) were placed at the vertex (V), located equidistantly between the caudal border of the occipital bone and intercanthus at midline, left mastoid (LM), right mastoid (RM), and on the dorsal midline at the level of the cranial aspect of second cervical vertebra (C2) for recording the BAER. The electrode at the mastoid area was placed caudal to the base of the ear in direct contact with the mastoid process. The electrode placed at the mastoid contralateral to the stimulated side served as the ground. Both ears were evaluated and BAER peaks were labeled, when visible, using Roman numerals I through V. Each BAER recording consisted of the average of a minimum of 400 responses over a 10 ms epoch. A simultaneous masking sound was used on the contralateral side with an offset of −30 decibels of normal hearing level (dBnHL). All BAER studies were performed in duplicate and 2 derivations were recorded simultaneously: (a) vertex to ipsilateral mastoid (V‐M) and (b) vertex to C2 (V‐C2).

Data recorded included latency and amplitude for peaks I and III measured in milliseconds and microvolts (μV), respectively; interpeak interval (IPI) for latency between peaks I‐V; and amplitude ratio by dividing peak V by peak I. Data from the baseline BAER test was compared to the BAER database from our institution acquired from healthy adult control horses for the identification of abnormalities. A BAER study was considered abnormal based on: (a) complete absence of identifiable BAER peaks (complete auditory loss); (b) increased peak latency; (c) decreased peak amplitude; or (d) combination of increased peak latency and decreased amplitude. The last 3 alterations were defined as partial auditory loss. Once baseline BAER was determined as normal or abnormal, subsequent BAER studies (after last dose of GENT and 30 days after last dose of GENT) were compared to the baseline BAER of each individual horse for the detection of alterations. Of particular importance was the determination of peak I latency because it is the first peak to be generated and dependent upon proper function of hair cells.^28^ Peak I latency delay of ≥2 ms from each horse's baseline BAER was considered abnormal.^29^

### 2.6 Bone conduction brainstem auditory evoked potentials

Using the same Nicolet VikingQuest machine, a bone conductor (VikingQuest) was placed and pressed at the level of the mastoid bone of the tested ear and used as the source of stimulus. Subcutaneous needle electrodes at the vertex, left and right mastoid, and C2 locations remained the same as those described for BAER. The testing sound consisted of 55 dBnHL vibration stimulus delivered through the bone conductor and a masking sound of 25 dBnHL air‐conducted stimulus delivered through an insert earphone to the contralateral ear. Higher stimulus intensities were not performed to avoid stimulus artifacts.^30^ An average of 400 responses in duplicates were performed using previously described derivations (V‐M, V‐C2). Because the variability of number and morphology of peaks in bone conduction (BC) studies, only presence or absence of peaks was recorded. Bone conduction was not performed at baseline if BAER was normal. The study only was done in the absence of detectable peaks based on the second BAER study to confirm sensorineural auditory loss and rule out an acquired conduction disorder.

### 2.7 Statistical analysis

**FIGURE 1:**
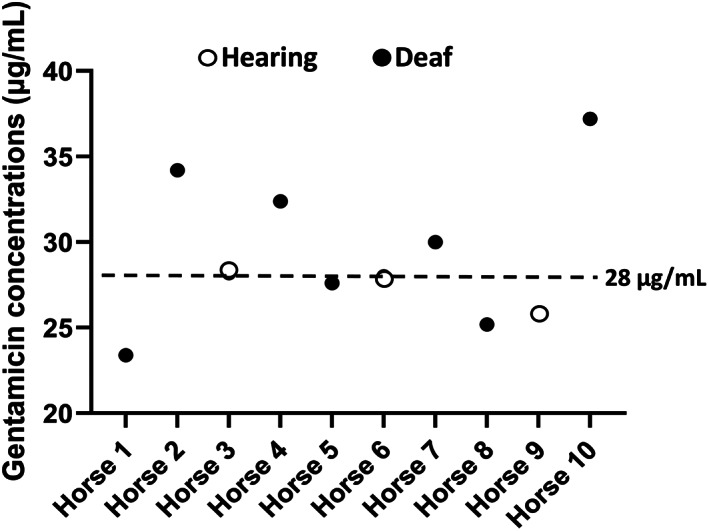
Scatter plot of peak GENT concentrations in 10 horses used to determine the cut‐off point for construction of the 2 × 2 table. Based on the scatter plot, peak concentrations were dichotomized as ≤28 μg/mL or >28 μg/mL (risk group). Horizontal dashed line = 28 μg/mL, open circles = Hearing, filled circles = Deaf

Data points measured were assessed for normality using the Shapiro‐Wilk test. Mean ± SD was reported when data were normally distributed whereas median (range) was reported when data were not normally distributed. Baseline BAER (first BAER) data was compared to our institutional BAER database for healthy adult horses to determine auditory status as normal or altered. Then, each horse's baseline BAER data served as its own control for subsequent BAER studies (BAER on days 7 and 37). Median (range) for peak latencies, interval, and amplitudes were calculated. Undetectable peaks were defined as complete auditory loss, and alterations in peak latency and amplitude as partial auditory loss. Associations between GENT peak concentrations and development of auditory loss and recovery vs no recovery of auditory function were analyzed using Fisher's exact test after construction of a 2 × 2 table. This association was investigated using a concentration above stated threshold. In addition to a small sample size available in our study, no data was available indicating GENT peak concentrations associated with auditory loss in horses. Therefore, a scatter plot (Figure 1) was constructed to visualize the distribution of GENT peak concentrations. The cut‐off for GENT peak concentrations associated with auditory loss or recovery in the 2 × 2 frequency table was determined using a scatter plot such that it divided the horses equally into 2 groups to avoid cells with zero counts in the frequency table. Based on the scatter plot, peak concentrations were dichotomized as ≤28 μg/mL or >28 μg/mL (risk group). Risk ratios and 95% confidence intervals (95% CI) were calculated using the 2 × 2 table. Risk ratios were considered significant if >1 or <1, with 95% CI excluding 1, and associated *P* < .05. Statistical software (GraphPad Prism, v8.4.3, San Diego, CA) was used and *P* < .05 was considered significant.

## 3 RESULTS

### 3.1 Animals

All horses had normal clinical examinations. Neurological examinations before first dose, after last dose (day 7), and 30 days after discontinuation of GENT administration identified no apparent abnormalities in behavior, mentation, cranial nerves (including auditory), or locomotion associated with vestibular disease. To assess for hearing, a loud clap by the examiner was made and all horses responded to the sound (startled or moved their ears). Further evaluation to assess for localization of sound was not performed. The examiner moved closer to the horse to detect audible tinnitus and none of the horses had apparent tinnitus. To further evaluate vestibular function, horses were blindfolded and allowed to stand or walk. None of the horses displayed vestibular deficits.

### 3.2 Clinical laboratory and gentamicin concentrations

Serum biochemistry panels at baseline and after last dose of GENT (day 7) identified no abnormalities. Serum creatinine concentrations remained within reference range (0.9‐2 mg/dL) and no significant difference was found between the baseline (mean 1.1 ± 0.1 mg/gL) and second (mean 1.2 ± 0.2 mg/dL) panels.

Mean trough and peak concentrations of GENT were <0.5 ± 0 μg/mL and 29.2 ± 4.3 μg/mL, respectively. When separating horses by group (hearing vs auditory loss), the peak concentrations were 27.3 ± 1.3 vs 30 ± 5 μg/mL for hearing and auditory loss, respectively. No association was found between peak GENT concentrations >28 μg/mL and development of auditory loss (relative risk, 1.33; 95% CI, 0.54, 3.66; *P* > .99) and outcome (recovery vs no recovery of auditory function; relative risk, 2.25; 95% CI, 0.56; 12.8; *P* = .49).

### 3.3 Brainstem auditory evoked responses

**FIGURE 2:**
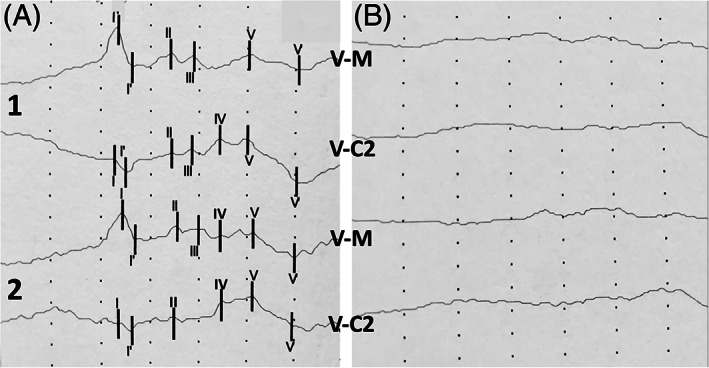
BAER tracings at 95 dBnHL from a mare with complete unilateral auditory loss (right ear). 2A = Pre‐GENT, 2B = Postlast dose of GENT on day 7. V‐M = vertex to mastoid, V‐C2 = vertex to C2, numbers 1 and 2 on left side of figure represent duplicates recorded from the right ear. Roman numbers represent detectable peaks. Each division represents 1 μV (vertical) and 1 ms (horizontal)

Horses had normal baseline BAER similar to that of our control database population (data not shown). Furthermore, no alterations were found between data from baseline and second BAER in 4 horses. However, abnormal BAER was detected in 6 horses. Three mares had unilateral abnormal BAER: 2 complete (right side, Figure 2) and 1 partial (left side) auditory loss. Three geldings had unilateral (right = 2, left = 1) partial auditory loss. Horses with partial auditory loss had increased peak latencies (peak I with 2‐3 ms delay from each horse's baseline). The contralateral ear in these 6 horses was not different from baseline data. Upon BAER testing 30 days after discontinuation of GENT, auditory loss was reversible in 4 horses with unilateral loss (complete = 2, partial = 2) and peak latencies and amplitudes returned to baseline values. Two horses remained partially deaf in 1 ear (2 ms delay from baseline), and 1 mare with previous auditory function on first 2 BAER studies was detected to have complete bilateral auditory loss. Table 1 shows summary baseline BAER data from 20 ears (10 horses), and data only from ears with partial auditory loss from the second BAER (ears = 4) and third BAER (ears = 2). The remainder of the ears for the second and third BAER were either unaltered from baseline (normal) or did not have detectable peaks (complete auditory loss) and such data are not shown in Table 1.

**TABLE 1:** BAER results

BAER #1: Baseline‐pre GENT (day 1)						
N = 20 ears	10 healthy adult horses within reference values					
95 dBnHL	LAT I (ms)	LAT V (ms)	IPI I‐V (ms)	AMP I (μV)	AMP V (μV)	Ratio V/I
V‐M	2.2 (2.2‐2.3)	5.5 (5‐5.9)	3.3 (2.7‐3.6)	1.3 (0.2‐2.4)	0.7 (0.4‐1)	0.6 (0.3‐3.7)
V‐C2	2.1 (2‐2.2)	5.6 (5‐5.9)	3.4 (2.8‐3.8)	0.5 (0.1‐0.8)	1.5 (0.8‐2.3)	2.9 (1.9‐6)

BAER #2: Last GENT (day 7)						
Auditory loss: Total 7/10 horses (detected BAER #2 = 6, BAER #3 = 1^a^)						
Data presented for ears with partial auditory loss only						
N = 4 ears	Auditory loss: N = 6/10 horses (C UNI = 2, P UNI = 4)					
95 dBnHL	LAT I (ms)	LAT V (ms)	IPI I‐V (ms)	AMP I (μV)	AMP V (μV)	Ratio V/I
V‐M	2.5 (2.4‐2.5)	5.7 (5.6‐5.8)	3.2 (3.1‐3.4)	1.1 (0.6‐1.5)	0.7 (0.6‐1)	0.7 (0.6‐1)
V‐C2	2.3 (2.3‐2.4)	5.8 (5.6‐5.8)	3.3 (3.3‐3.4)	0.4 (0.2‐0.7)	0.8 (0.4‐1.1)	2.5 (0.5‐3.8)

BAER #3: 30D after last GENT (day 37)						
N = 2 ears	Auditory loss: N = 3/10 horses (persistent = 2 P UNI, new = 1^a^ C BI); recovered = 4					
95 dBnHL	LAT I (ms)	LAT V (ms)	IPI I‐V (ms)	AMP I (μV)	AMP V (μV)	Ratio V/I
V‐M	2.4 (2.4)	5.8 (5.7‐5.9)	3.4 (3.3‐3.5)	1.1 (0.9‐1.3)	0.4 (0.3‐0.5)	0.4 (0.3‐0.4)
V‐C2	2.3 (2.3)	5.5 (5.4‐5.6)	3.2 (3.1‐3.3)	0.8 (0.8)	1.3 (1‐1.6)	1.7 (1.3‐2)

*Notes*: Table depicting median (range) for peak latencies, interpeak interval, amplitudes, and amplitude ratios at 95 dBnHL. Baseline BAER data for 10 horses (20 ears) is presented. BAER #2 and #3 only present data from ears with partial auditory loss (increased peak I latency of ≥2 ms from baseline), rest of ears were normal or completely deaf (data not shown). V‐M and V‐C2, BAER #1: baseline = BAER prior to first GENT dose (day 1); BAER #2 = BAER after last GENT dose (day 7); BAER #3 = BAER 30 days after last GENT dose (day 37).

Abbreviations: AMP, amplitude in microvolts (μV); BI, bilateral; C, complete auditory loss; IPI, interpeak interval (ms); LAT, latency in milliseconds; P, partial auditory loss; UNI, unilateral.

^a^ New case detected on BAER #3.

### 3.4 Bone conduction brainstem auditory evoked responses

Bone conduction was performed in 2 horses with complete unilateral auditory loss based on BAER on day 7 to rule out an acquired conduction problem. Peaks were not detected in these 2 horses, ruling out a conduction disorder and further supporting sensorineural auditory loss. Bone conduction was not performed in the mare that developed complete bilateral auditory loss based on the last BAER study (day 37).

## 4 DISCUSSION

We showed an association between sensorineural auditory loss and administration of GENT at the recommended route and dosage of administration for 7 consecutive days. The combination of physical examination (clean ears with no apparent pathology) and comparison of BAER at baseline and after the last dose of GENT supported sensorineural auditory loss. Furthermore, the combination of BAER and BC confirmed sensorineural auditory loss in 2 horses with complete absence of BAER. Auditory loss developed in 7 of 10 horses in our study. Unilateral sensorineural auditory loss was detected in 6 of 10 horses at the BAER performed after the last GENT dose and was partial in 4 and complete in 2 cases. Only 1 mare developed complete bilateral auditory loss detected on BAER performed 30 days after discontinuation of GENT. Recovery of auditory function occurred in 4 of 7 horses. Gentamicin peak concentrations were not associated with development of auditory loss or recovery of function. Although our sample size was small, no breed or sex predisposition to susceptibility of auditory loss associated with GENT administration was identified. Tinnitus was not detected on physical and neurological examination. However, further diagnostic investigation for tinnitus was not performed. None of the horses developed signs of vestibular disease even when challenged by blindfolding. Furthermore, clinical and laboratory evidence of nephrotoxicity was not detected.

Gentamicin is a widely used cost‐effective antimicrobial agent in equine medicine for which an IV dosage of 6.6 mg/kg has been used routinely.^9^, ^11^ However, 2 recent studies have proposed use of higher dosages of GENT administered IV to target susceptible bacteria in diseased horses.^27^, ^31^ One study suggested an initial once‐daily dosage >7.7 mg/kg of GENT IV to achieve desired peak concentrations of >32 μg/mL to treat common bacterial isolates and detect possible toxic concentrations based on trough concentrations <2 μg/mL.^27^ Another study suggested an IV dosage of 10 mg/kg to reach or exceed peak concentrations of 20 μg/mL, which is 10 times the minimum inhibitory concentration (MIC) of susceptible bacteria.^31^ However, safety and toxic effects of higher doses were not investigated in either study.^27^, ^31^ Serum biochemical analysis to assess kidney function and therapeutic drug monitoring are essential to determine proper dosing regimens and avoid possible adverse effects such as nephro‐, vestibulo‐, and cochleotoxicity. A GENT study in 6 healthy adult ponies using 5 mg/kg dosage q8h IM for 2 different durations (7 and 14 consecutive days, respectively, in 3 ponies for each group) showed no alterations in BAER.^7^ Mean GENT peak concentrations in ponies ranged from 13.57 to 25.26 μg/mL and 15.18 to 16.65 μg/mL for the 7 and 14 day groups, respectively.^7^ In our study, mean GENT peak concentrations were 29.2 μg/mL for all 10 horses. Upon grouping horses under those that developed auditory loss (N = 7) as compared to those that retained auditory function (N = 3) throughout the study, mean GENT peak concentrations were 30 and 27.3 μg/mL, respectively. Although an association between specific GENT peak concentrations and development of auditory loss was not found in our study, it is possible that differences in the results from both studies (horses vs ponies) was because of differences in GENT peak concentrations (higher in our horses). This assumption needs further investigation with a larger population of horses.

Cochleotoxicity and vestibulotoxicity have been reported to occur in 2% to 25% and 15% of human patients after AG therapy, respectively.^32^ Both ototoxic effects have been reported as irreversible in humans.^33^ Depending on the frequencies used for BAER testing, auditory loss has been documented in up to 47% of human patients with a history of AG treatment.^34^ The prevalence is likely underestimated because of lack of routine patient monitoring with audiometric techniques. Possible ototoxic effects were investigated in our study by neurological and BAER examinations. Neurological examination included evaluation of hearing and vestibular function at the beginning and end of GENT administration and 30 days after the end of the study. Hearing was evaluated by the horses' response to environmental auditory stimuli and clapping hands. All horses in our study showed a response to auditory stimuli such as turning the head, moving the ears, or getting startled by sound. Evaluation of sound localization based on neurological examination was not attempted because the results could be variable. Audible tinnitus was not clinically apparent in these horses. Vestibular function was evaluated by observation of ocular position, presence of physiologic nystagmus, lack of pathologic nystagmus, head and body position, and gait. Horses also were blindfolded to further assess vestibular function because visual compensation is important if vestibular disease is present. No alterations in head and body position, balance, or gait were noted upon blindfolding.

Brainstem auditory evoked responses evaluate the integrity of the auditory pathway from its peripheral to central parts.^28^, ^30^ Bone conduction bypasses the external and middle ear by conducting sound through bone (mastoid) and detected by the cochlea in the inner ear.^30^ Therefore, conductive disorders such as those caused by external ear canal obstruction by debris, cerumen, fluid, foreign body, ticks, polyps, neoplasia, otosclerosis, trauma, tympanic membrane rupture, and middle ear disorders including congenital anomalies will result in normal BC with altered or absent BAER.^2^, ^35^, ^36^ Sensorineural auditory loss can be peripheral (hair cells within the cochlea, spiral ganglion, or cochlear nerve) or central (brainstem), depending on which anatomical part of the auditory pathway is dysfunctional.^29^, ^37^ Aminoglycoside ototoxicity resulting in sensorineural auditory loss from hair cell dysfunction and degeneration has been well‐documented in human patients.^19^ Similarly, we assumed that horses in our study developed sensorineural auditory loss induced by GENT administration. We recognize that BAER testing might not be feasible or available to monitor auditory function when using AG.

Sensorineural auditory loss can occur during or within days to weeks after systemic AG administration as a result of hair cell dysfunction.^19^ Similarly, 60% of our horses developed auditory loss while on GENT treatment, whereas 1 mare developed auditory loss days after discontinuation of treatment. The exact time at which this loss occurred is unknown because horses were not evaluated between the day of the last dose and 30 days after. Hair cell dysfunction can be further investigated by otoacoustic emissions and cochlear microphonics testing which were not performed in our study.^30^, ^38^, ^39^, ^40^ Duration of GENT administration might vary from a single dose used as premedication for elective surgical procedures, a few days after surgery, to several days to weeks to treat infections such as sepsis, pleuropneumonia, osteomyelitis, and others.^41^, ^42^, ^43^ Development and time at onset of hair cell dysfunction in human patients have been proposed to be dose‐ and duration‐dependent.^19^ Here, we investigated the most commonly used route, dosage, and dosing interval in horses for 7 consecutive days. It is unknown at present if other GENT dosing protocols (eg, dosage, interval) and duration of administration (single dose vs multiple doses over days to weeks), age, and state of health or disease might play a role in the development of sensorineural auditory loss.

Aminoglycosides‐induced auditory dysfunction is most often bilateral and can be complete or partial.^18^ In our study, auditory loss was unilateral in 6 of 6 horses (complete = 2, partial = 4) with most being affected on the right side (N = 4 of 6). This observation was unlikely to represent GENT targeting preferentially to the right side and more likely to reflect small sample size. It is unclear if extension of treatment would have affected both ears. There were no apparent or known predisposing factors in these horses that could have increased the risk of becoming deaf in 1 ear, because baseline BAER was normal. Aminoglycosides‐induced cochleotoxicity is irreversible in humans and other species.^19^ However, in our study, 4 horses recovered auditory function as evidenced by follow‐up BAER 30 days after discontinuation of treatment. This finding presumably reflects malfunction rather than degeneration of hair cells during GENT administration.

In our study, no breed or sex predilection to the susceptibility of auditory loss was identified. However, a larger number of horses of both sexes will be needed to further explore this assumption. This result is similar to reports of human patients for whom sex predilection has not been reported.^44^ A genetic basis for susceptibility to AG‐induced ototoxicity has been identified in humans.^18^, ^45^, ^46^, ^47^, ^48^ Impairment of RNA translation within mitochondria linked to mutations in the 12S rRNA gene (nucleotide A1555G) increases structural similarity to bacterial rRNA (16 seconds rRNA) which promotes binding of AG to mutated mitochondrial rRNA.^18^, ^45^ This results in the production of reactive oxygen species and oxidative stress mediated by an iron‐AG complex resulting in apoptosis and necrosis of the hair cells of the cochlea, marginal cells, and *stria vascularis*.^18^ This mutation has been reported in 17% to 33% of patients with AG ototoxicity.^49^ Other mutations also have been described as increasing AG susceptibility.^18^ Therefore, a genetic predisposition also might play a role in the susceptibility to AG toxicity in horses. Antioxidants such vitamin E, silymarin, some salicylates, and iron chelators have been used in attempts to provide otoprotection by minimizing degeneration of hair cells associated with AG in humans, mice, and dogs with variable results.^18^, ^19^, ^50^, ^51^ Given the risk of AG‐induced ototoxicity in horses based on our study, consideration and investigation of concurrent antioxidant therapy might be warranted.

Nephrotoxicity is a known possible toxic effect of AG treatment which is usually reversible.^52^, ^53^ This adverse effect has been reported in approximately 25% of humans on AG treatment and results from direct tubular toxicity from mitochondrial damage, decreased glomerular filtration rate, and decreased renal blood flow.^53^ Risk factors identified include advanced age, liver or kidney disease, and dehydration, among others.^53^ Nephrotoxicity associated with GENT administration has been reported in horses, particularly younger animals.^54^, ^55^ Transient increases in serum creatinine concentration and urine gamma‐glutamyl transferase activity have been reported in horses with the use of GENT.^56^ Although horses in our study showed mild increases in serum creatinine concentration after the last dose of GENT, these concentrations remained within the reference range and the increase was not statistically significant. Furthermore, horses appeared clinically healthy days after completion of the study.

Our study had some limitations. Susceptibility to GENT‐induced ototoxicity by breed, sex, and age was not determined because of the low number of horses enrolled. Furthermore, only young to early middle age adult horses were included. Therefore, it is unknown if GENT susceptibility might be age‐dependent (neonates and foals vs geriatric horses). Susceptibility based on states of health and disease were not investigated in our study because all horses were healthy. Therefore, it is unknown if GENT peak concentrations might vary with the phase of treatment of Gram‐negative infections resulting in GENT being more or less available to target hair cells. For instance, higher GENT doses required to target specific pathogens might decrease the available GENT compounds that could target hair cells whereas similar doses might pose a higher risk for healthy horses because there is no competition for possible targets. Different dosing protocols including single vs multiple, different doses and intervals, and different duration were not investigated. Daily or weekly BAER testing would have been useful to determine approximate time of development of auditory dysfunction. Furthermore, our study did not include long‐term follow‐up (beyond 30 days after the last dose) to investigate if auditory function would be reversible in the remaining 3 horses. Despite these limitations, our study provides evidence that GENT can induce auditory loss in horses.

## 5 CONCLUSIONS

Sensorineural auditory loss is a potential risk when using GENT in horses. In our study, an IV dosage of 6.6 mg/kg for 7 days caused sensorineural auditory loss in 7 of 10 healthy adult horses. Vestibulo‐ and nephrotoxicity were not detected at this dosage and duration. Auditory loss can develop during administration or days after discontinuation of treatment. This dysfunction can be partial or complete, unilateral or bilateral, and in some cases irreversible. Auditory loss if complete can impair the behavior, performance, and ultimately quality of life of the horse. Furthermore, horses might get startled and pose a safety risk for those involved in their care and training. Additional studies are needed to investigate if the risk of GENT‐induced auditory loss is dependent on age, breed, sex, different dosing protocols and states of health or disease.

## CONFLICT OF INTEREST DECLARATION

Munashe Chigerwe serves as Associate Editor for the Journal of Veterinary Internal Medicine. He was not involved in review of this manuscript. No other authors have a conflict of interest.

## OFF‐LABEL ANTIMICROBIAL DECLARATION

Authors declare no off‐label use of antimicrobials.

## INSTITUTIONAL ANIMAL CARE AND USE COMMITTEE (IACUC) OR OTHER APPROVAL DECLARATION

Approved by the IACUC of University of California, Davis, number 22043.

## HUMAN ETHICS APPROVAL DECLARATION

Authors declare human ethics approval was not needed for this study.
